# Appropriate NH_4_^+^/NO_3_^–^ Ratio Triggers Plant Growth and Nutrient Uptake of Flowering Chinese Cabbage by Optimizing the pH Value of Nutrient Solution

**DOI:** 10.3389/fpls.2021.656144

**Published:** 2021-04-30

**Authors:** Yunna Zhu, Baifu Qi, Yanwei Hao, Houcheng Liu, Guangwen Sun, Riyuan Chen, Shiwei Song

**Affiliations:** ^1^College of Horticulture, South China Agricultural University, Guangzhou, China; ^2^Henry Fok College of Biology and Agriculture, Shaoguan University, Shaoguan, China

**Keywords:** ammonium to nitrate ratio, xylem exudate, nitrogen efficiency, plasma membrane H^+^-ATPase, flowering Chinese cabbage

## Abstract

Compared with sole nitrogen (N), the nutrition mixture of ammonium (NH_4_^+^) and nitrate (NO_3_^–^) is known to better improve crop yield and quality. However, the mechanism underlying this improvement remains unclear. In the present study, we analyzed the changes in nutrient solution composition, content of different N forms in plant tissues and exudates, and expression of plasma membrane (PM) H^+^-ATPase genes (*HAs*) under different NH_4_^+^/NO_3_^–^ ratios (0/100, 10/90, 25/75, 50/50 as control, T1, T2, and T3) in flowering Chinese cabbage. We observed that compared with the control, T1 and T2 increased the economical yield of flowering Chinese cabbage by 1.26- and 1.54-fold, respectively, whereas T3 significantly reduced plant yield. Compared with the control, T1–T3 significantly reduced the NO_3_^–^ content and increased the NH_4_^+^, amino acid, and soluble protein contents of flowering Chinese cabbage to varying extents. T2 significantly increased the N use efficiency (NUE), whereas T3 significantly decreased it to only being 70.25% of that of the control. Owing to the difference in N absorption and utilization among seedlings, the pH value of the nutrient solution differed under different NH_4_^+^/NO_3_^–^ ratios. At harvest, the pH value of T2 was 5.8; in the control and T1, it was approximately 8.0, and in T3 it was only 3.6. We speculated that appropriate NH_4_^+^/NO_3_^–^ ratios may improve N absorption and assimilation and thus promote the growth of flowering Chinese cabbage, owing to the suitable pH value. On the contrary, addition of excessive NH_4_^+^ may induce rhizosphere acidification and ammonia toxicity, causing plant growth inhibition. We further analyzed the transcription of PM H^+^-ATPase genes (*HAs*). *HA1* and *HA7* transcription in roots was significantly down-regulated by the addition of the mixture of NH_4_^+^ and NO_3_^–^, whereas the transcription of *HA2*, *HA9* in roots and *HA7*, *HA8*, and *HA10* in leaves was sharply up-regulated by the addition of the mixture; the transcription of *HA3* was mainly enhanced by the highest ratio of NH_4_^+^/NO_3_^–^. Our results provide valuable information about the effects of treatments with different NH_4_^+^/NO_3_^–^ ratios on plant growth and N uptake and utilization.

## Introduction

Ammonium (NH_4_^+^) and nitrate (NO_3_^–^) are two main nitrogen (N) forms that can be absorbed and utilized by plants (Xu et al., 2012), and they have an important effect on crop growth and quality (Hachiya and Sakakibara, 2017). In most aerated soils, the major N form is NO_3_^–^, whereas NH_4_^+^ is dominant in acidic and/or anaerobic soils (Zhu et al., 2011). The roots of most plants prefer the uptake of NH_4_^+^ over NO_3_^–^ in micromolar concentrations (Hachiya and Sakakibara, 2017) owing to the lower energy costs associated with the absorption and assimilation of NH_4_^+^ than those of NO_3_^–^ (Guo et al., 2007); on the contrary, NH_4_^+^ often causes ammonium toxicity at millimolar concentrations (Britto and Kronzucker, 2002). Studies have revealed that co-provision of NH_4_^+^ and NO_3_^–^ nutrition significantly stimulated plant growth in strawberry (Tabatabaei et al., 2008), mini Chinese cabbage (Hu et al., 2015), flowering Chinese cabbage (Song et al., 2017), and Chinese kale (Zhu et al., 2018) in comparison with the addition of NH_4_^+^ or NO_3_^–^ alone. Moreover, the mixture of NH_4_^+^ and NO_3_^–^ increased the content of soluble sugars, soluble proteins, and vitamin C in plants (Tabatabaei et al., 2008) and reduced the content of nitrates (Song et al., 2017; Zhu et al., 2018). Therefore, addition of a mixture containing appropriate ratios of NH_4_^+^ and NO_3_^–^ is beneficial to plant growth and development. Several studies have reported that NH_4_^+^ and NO_3_^–^ can interact with each other when co-supplying two N forms (Kronzucker et al., 1999; Hachiya and Sakakibara, 2017; Zhu et al., 2020).

NO_3_^–^ and NH_4_^+^ are absorbed by plant cells from the soil via specific proteins called nitrate transporters (NRTs) and ammonium transporters (AMTs), respectively (Nacry et al., 2013). In *Arabidopsis*, NRTs consist of nitrate transporter 1/peptide transporter family (NRT1/PTR), NRT2, chloride channels (CLC), and slow anion channel-associated 1 homologs (SLAC1/SLAH), which are involved in low or high affinity uptake, xylem loading, and ion efflux of NO_3_^–^ (Krapp et al., 2014). AMTs include two subfamilies, AMT1 and AMT2. Recent research has mainly focused on AMT1, which takes part in the absorption and transport of NH_4_^+^ (Yuan et al., 2007; Straub et al., 2017). Both NRTs and AMTs are located in the plasma membrane (PM) (Sperandio et al., 2014). PM H^+^-ATPase (PM H^+^-ATPase, EC 3.6.1.3.) is a proton pump that is necessary to promote cell growth and ion fluxes across the PM (Sperandio et al., 2020). NH_4_^+^ transmembrane transport is controlled by electrochemical potential inside and outside the cell membrane, and this process does not require energy (Wang et al., 1993). NH_4_^+^ uptake can lead to depolarization of the cell membrane and can enhance the activity of proton pumps (Schubert et al., 1990); in contrast, NO_3_^–^ transmembrane transport is an active transport process which requires energy and H^+^ provided by PM H^+^-ATPase (Gaxiola et al., 2007).

Therefore, either NH_4_^+^ or NO_3_^–^ uptake is related to the activity of proton pumps. H^+^-ATPase, which is an important functional protein of the PM, is called the “master enzyme” in plants (Michelet and Boutry, 1995). PM H^+^-ATPase is responsible for establishing a proton electrochemical gradient in the membrane energization used for solute transport, and it controls the major transport processes in plants such as root nutrient uptake, cell elongation, xylem and phloem loading, stomatal aperture, and cellular pH regulation (Duby and Boutry, 2009; Zeng et al., 2012). In addition, proton pump in PM is responsible for other important physiological functions, such as stomatal aperture (Janicka-Russak, 2011).

In plants, PM H^+^-ATPases (autoinhibited H^+^-ATPases), which form one subfamily of P-type ATPases, are encoded by a multi-gene family (Arango et al., 2003). There are 12 members of this family in *Arabidopsis thaliana* (AHA1–AHA12), nine in *Nicotiana plumbaginifolia* (PMA1–PMA9), and ten in *Oryza sativa* (OSA1–OSA10), and they are classified into five subfamilies (Arango et al., 2003; Pii et al., 2014). The members of subfamilies I and II are expressed throughout the plant with different intensity in different organs (Gaxiola et al., 2007). For instance, Ueno et al. (2005) reported that in *Arabidopsis*, *AHA1*, *2*, *3*, *5*, *7*, *8*, *10*, and *11* were expressed in green leaves, and *AHA1*, *2*, *4*, *7*, *8*, *10*, and *11* were expressed in roots. AHA1 is a housekeeping protein found all over the plant (Santi and Schmidt, 2009), whereas AHA2 plays major roles in root metabolism (Gaxiola et al., 2007; Hoffmann et al., 2019). However, subfamilies III, IV, and V are not highly expressed under normal conditions and are expressed in specific tissues (Arango et al., 2003). *AHA6* and *AHA9* are expressed only in the anthers (Gaxiola et al., 2007). The expression of PM H^+^-ATPase isoforms is affected by N forms, and *OSA2* and *OSA7* in *O. sativa* are strongly induced in response to N resupply and may be involved in N uptake (Sperandio et al., 2011). The expression and activity of PM H^+^-ATPase are affected by other nutrient elements (i.e., P and K^+^) (Yuan et al., 2017).

As a variety of Chinese cabbage (*Brassica rapa*), flowering Chinese cabbage (*Brassica campestris* L. ssp. *chinensis* var. *utilis* Tsen et Lee) is an important leaf vegetable in South China whose product organs are leaves and stalks (Song et al., 2012). In previous study, we have shown that a mixture of NH_4_^+^ and NO_3_^–^ is more beneficial to the growth and quality of flowering Chinese cabbage than a single N source and that it improves plant NUE (Song et al., 2012). Furthermore, ammonium transporter 1.2 (AMT1.2) mediates the interaction of NH_4_^+^ and NO_3_^–^ under controlled conditions when the pH value is adjusted to 5.8 (Zhu et al., 2020). It is generally known that the absorption of NH_4_^+^ can induce net H^+^ release and acidify the rhizosphere (Schubert and Yan, 1997); on the contrary, the absorption of NO_3_^–^ can increase H^+^ uptake through a H^+^ co-transport system in PM and alkalize the rhizosphere (Zeng et al., 2012). However, our understanding of the mechanisms underlying the plant uptake of different forms of N and their transport and assimilation depending on different ratios of NH_4_^+^ and NO_3_^–^ is limited.

In the present study, we examined the characteristics of different N forms in flowering Chinese cabbage seedlings and nutrient solutions as well as the N form composition of plant exudates. Furthermore, we analyzed the expression of PM H^+^-ATPase genes in flowering Chinese cabbage in response to different NH_4_^+^/NO_3_^–^ ratios using quantitative real-time PCR.

## Materials and Methods

### Plant Material and Treatments

The experiment was carried out in the greenhouse of South China Agricultural University, Guangzhou, Guangdong Province. Flowering Chinese cabbage seeds (cultivar ‘Youlv 501’) were provided by Guangzhou Academy of Agriculture Science (Guangdong Province). Plug seedlings were used with perlite as the growth medium. Three consistent seedlings with developed third true leaves were selected and transplanted into plastic bucket filled with 5.5 L of nutrient solution. There were 12 replications in each treatment which was arranged in a randomized complete block design. All nutrient solutions were aerated for 15 min per hour using a controlled pump. Hoagland-Snyder formula (3/4 of the dose) was used as basic and contrast nutrient solution, with 3.75 mmol L^–1^ KNO_3_, 3.75 mmol L^–1^ Ca(NO_3_)_2_, 0.75 mmol L^–1^ KH_2_PO_4_, 2.1 mmol L^–1^ MgSO_4_. In the nutrient solutions with different NH_4_^+^/NO_3_^–^ ratios nutrient solution, NH_4_^+^ was supplied by NH_4_Cl. KCl or CaCl_2_ was added to maintain consistent concentrations across the treatments. During the entire growth period, the nutrient solution was not replaced, and it was supplemented with deionized water every 3 days until reaching the original volume. Electrical conductivity and pH were measured during the experiment.

### Parameter Measurements

Plant seedlings were harvested randomly when they reached marketable maturity. Their root or shoot weight was measured; the fresh weight of the product organ (flower stalk above the 4th node) was called commercial yield. The growth rate was calculated from the difference in fresh weight before and after sampling divided by the number of days. Roots, stems, and leaves of the seedlings were harvested, immediately frozen in liquid N_2_, and stored at −80°C.

The contents of NH_4_^+^ and NO_3_^–^ were analyzed as described by Ivančič and Degobbis (1984) and Patterson et al. (2010), respectively. Amino acid content was measured as described by Inada et al. (2006), and leucine was used as a standard for amino acid content estimation. Soluble protein content was determined with bovine serum albumin as the standard (Bradford, 1976). Total N concentration was determined with an auto-analyzer (Kjeltec 2300 Analyzer Unit, Foss Tecator, Sweden) as described by Avery and Rhodes (1990), and total N concentration was multiplied by the dry weight of the whole plant to calculate N accumulation. As described by Eckstein and Karlsson (2001), N loss (NL), N loss rate (NLR), mean residence time of N (MRT), N productivity (NP), and NUE were calculated using the following formulas. NL =[(NS_*applied*_−NS_*remain*_)−(*N*_*harvest*_−*N*_*transplant*_)×*n*]/ *n*; NLR=NL/(NS_*applied*_−NS_*remain*_); MRT=(*N*_*harvest*_−*N*_*transplant*_)/ [(lnN_*harvest*_−lnN_*transplant*_)×(NL/t)]; NP=[(*W*_*harvest*_−*W*_*transplant*_)/ (*T*_*harvest*_−*T*_*transplant*_)]×[(lnN_*harvest*_−lnN_*transplant*_)/(*N*_*harvest*_−*N*_*transplant*_)]; NUE=NP×MRT. In the formulas above, W is the dry weight of the plant; T is the sampling time; N is the amount of N absorbed by seedlings; NS is the amount of N in the nutrient solution; n is the number of seedlings in each hydroponic bucket; and t is the number of days in the whole growth period. Total phosphorus (P) and potassium (K) concentrations were determined through a modified molybdenum blue procedure at 660nm using a spectrophotometer (UV-1800, Shimadzu, Japan) and through atomic absorption spectrophotometry (AA-6800, Shimadzu, Japan) (Westerman, 1990), then their accumulation was calculated by multiplying bythe dry weight.

### The Composition of Xylem Exudates of Flowering Chinese Cabbage in Response to Different NH_4_^+^/NO_3_^–^ Ratios

Flowering Chinese cabbage seedlings were cultured as described in Section “Plant Material and Treatments.” After pre-culturing for 5 days, the seedlings were transferred to an N-free Hoagland solution for 2 days. Three consistent seedlings were selected and transplanted in a barrel containing 5.5 L of the nutrient solution. Each treatment had six replications. Seedling exudates were collected after 2 weeks as described by Kehr et al. (2005) to analyze the intensity of xylem exudates and stored at −80°C to measure the content and flux of NO_3_^–^, NH_4_^+^, free amino acid, and soluble protein. The activity of roots was measured by using 2, 3, 5-tetraphenyltetrazolium chloride (TTC) as described by Yi et al. (2007).

### Quantitative Real-Time Polymerase Chain Reaction (qPCR)

Total RNA was extracted from the samples using an Eastep^®^ Super Total RNA Extraction Kit (Promega, Beijing, China), after which it was reverse transcribed using a GoScript^TM^ Reverse Transcription Mix (Promega, Beijing, China). The qPCR was performed in a CFX Connect Real-Time PCR System (Bio-RAD, CA, United States) using SYBR^®^ Premix Ex Taq^TM^ (TaKaRa Bio, Tokyo, Japan). The primer pairs were listed in Supplementary Table 1. *Actin2* and *GAPDH* were used as internal controls. Three biological replicates were used to calculate relative gene expression levels by the 2^–ΔΔ*CT*^ method (Livak and Schmittgen, 2002).

### Data Analysis

The data were analyzed by one-way analysis of variance (ANOVA) using the software package SPSS 19.0 (SPSS Incorporation, Chicago, IL, United States), and the differences among treatments were compared using the least-significant difference (LSD) test with a significance level of *P* < 0.05. Figures were made using SigmaPlot v11.1 (Jandel Scientific Software, San Rafael, CA, United States).

## Results

### Effect of Different NH_4_^+^/NO_3_^–^ Ratios on the Growth and Biomass of Flowering Chinese Cabbage Seedlings

**FIGURE 1:**
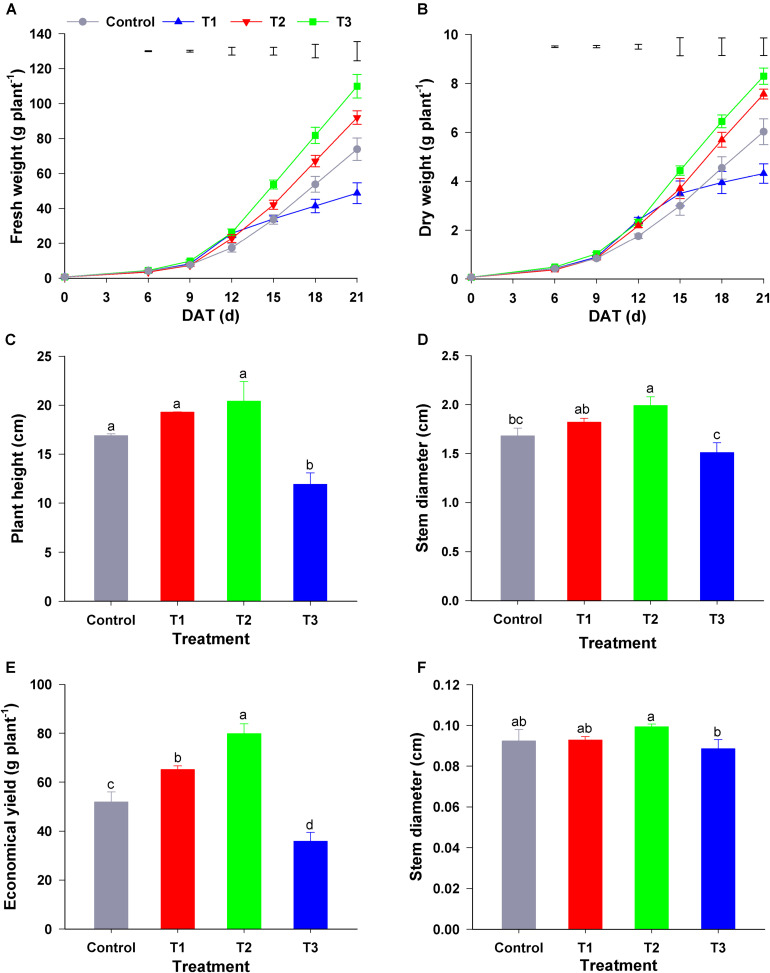
Effect of different NH_4_^+^/NO_3_^–^ ratios on the growth of flowering Chinese cabbage. **(A)** Dynamic changes in fresh weight; **(B)** dynamic changes in dry weight; **(C)** plant height at the harvest stage (after 21 days of treatment); **(D)** stem diameter at the harvest stage; **(E)** the ratio of root to shoot fresh weight at the harvest stage; and **(F)** economical plant yield at the harvest stage. Control = 0/100, T1 = 10/90, T2 = 25/75, T3 = 50/50. The data represent mean ± SE (*n* = 6). The vertical ruler in panels **(A,B)** represents the LSD_0_._05_ of the average values among the treatments. Different letters in (**C–F)** indicate significant differences at *P* < 0.05.

As shown in Figure 1, different NH_4_^+^/NO_3_^–^ ratios influenced the growth of flowering Chinese cabbage seedlings markedly. T1 (NH_4_^+^/NO_3_^–^ = 10/90), T2 (NH_4_^+^/NO_3_^–^ = 25/75), and T3 treatment (NH_4_^+^/NO_3_^–^ = 50/50) significantly increased the biomass of seedlings during the early growth stage (0–12 days after treatment) compared with the control (NH_4_^+^/NO_3_^–^ = 0/100) (Figure 1A). In contrast to the effect of other treatments, compared to that in the control, T3 significantly decreased the fresh and dry plant weight at the late stage of treatment (15–21 days) (Figure 1B). At the harvest stage of flowering Chinese cabbage (21 days), compared with that in the control, the height of flowering Chinese cabbage in T1 and T2 was increased by 1.14, and 1.21 times, respectively, whereas that in T3 was significantly decreased (Figure 1C). Similarly, the stem diameter was the highest in T2 and lowest in T3 (Figure 1D). Owing to a higher concentration of NH_4_^+^, T3 significantly inhibited the root growth of flowering Chinese cabbage, and these plants had the lowest root to shoot ratio (Figure 1E). Consistent with the changes in biomass, the economic yield was the highest in T2, being 1. 53-, 1. 17-, and 2.41-fold higher than that in the control, T1, and T3, respectively, whereas it was the lowest in T3, being only 69.10% that of the control (Figure 1F).

To study the influence of different NH_4_^+^/NO_3_^–^ ratios on seedling growth, we analyzed the growth rate of flowering Chinese cabbage throughout the experiment. During the period of 0–9 days after treatment, the growth rate of seedlings was the highest in T2, whereas there was no significant difference among the other three treatments (Table 1). During the period of 6–15 days, the growth rate was still the highest in T2, followed by T1, and it was the lowest in control and T3, which had no significant difference. Similarly, during the period of 15–21 days, the growth rate was still the highest in T2, with no significant difference between T2 and T1; the growth rate of control plants was significantly higher than that of plants in T3. In summary, at 0–15 days, there was no significant difference between the growth rate of seedlings in the control and T3 group; at 0–21 days, the growth rate was the lowest in T3: it was only 63.16, 48.25, and 41.13% that of the control, T1, and T2, respectively. This indicated that the highest ratio of NH_4_^+^/NO_3_^–^ (NH_4_^+^/NO_3_^–^ = 50/50) did not inhibit the growth of flowering Chinese cabbage seedlings at the early stage (at 0–15 days); but it significantly inhibited the growth since 15 days after treatment, its growth rate was only 36.57% of the control at 15–21 days.

**TABLE 1:** Effects of different NH_4_^+^/NO_3_^–^ ratios on the growth rate of flowering Chinese cabbage (g d^–1^ plant^–1^).

Treatments	NH_4_^+^/NO_3_^–^	0–9 days	6–15 days	15–21 days	0–15 days	0–21 days
Control	0/100	0.77 ± 0.06 b	4.35 ± 0.34 c	6.70 ± 0.64 b	2.20 ± 0.17 c	3.49 ± 0.31 c
T1	10/90	0.74 ± 0.06 b	5.80 ± 0.34 b	8.32 ± 0.22 a	2.76 ± 0.17 b	4.35 ± 0.19 b
T2	25/75	1.01 ± 0.07 a	7.32 ± 0.32 a	9.37 ± 0.69 a	3.53 ± 0.17 a	5.20 ± 0.32 a
T3	50/50	0.84 ± 0.05 b	4.31 ± 0.21 c	2.45 ± 0.70 c	2.23 ± 0.11 c	2.29 ± 0.28 d

*Control = 0/100, T1 = 10/90, T2 = 25/75, T3 = 50/50. The data represent mean ± SE (n = 3). Different letters indicate significant differences at P < 0.05.*

Consequently, we concluded that lower ratios of NH_4_^+^/NO_3_^–^ could promote the growth of flowering Chinese cabbage, among which the effect of T2 (NH_4_^+^/NO_3_^–^ = 25/75) was the strongest; the highest ratio of NH_4_^+^/NO_3_^–^ (NH_4_^+^/NO_3_^–^ = 50/50) could inhibit plant growth, especially at the later stage.

### N Content and the Distribution of Different N Forms in Flowering Chinese Cabbage Plants Under Different NH_4_^+^/NO_3_^–^ Ratios

**FIGURE 2:**
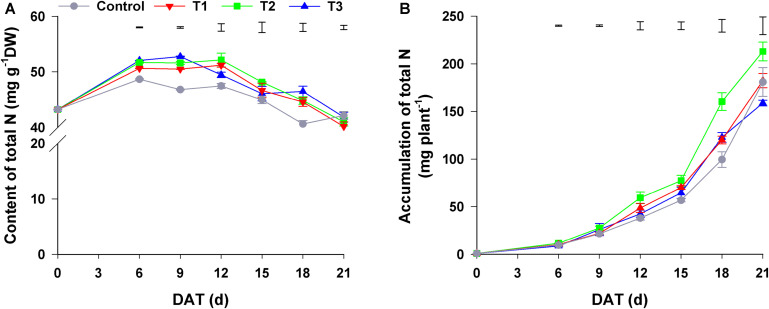
Effect of different NH_4_^+^/NO_3_^–^ ratios on the content **(A)** and accumulation **(B)** of total N during the growth period of flowering Chinese cabbage. Control = 0/100, T1 = 10/90, T2 = 25/75, T3 = 50/50. DW, dry weight; DAT, days after treatment. The data represent mean ± SE (*n* = 3). The vertical ruler represents the LSD_0_._05_ of the average values among the treatments.

As shown in Figure 2A, the total N content of flowering Chinese cabbage increased at first and then decreased during the growth period. The total N content of plants in T1–T3, among which there was no significant difference, was higher than that of control plants. Regarding the accumulation of total N in plants, it was the highest in plants in T2 throughout the entire growth period, especially at the late stage of growth (i.e., 18–21 days); control plants had the lowest total N in the period from 0 to 18 days of growth, whereas plants in T3 had the lowest total N at 21 days (only 74.56% of that of plants in T2) (Figure 2B). Besides the total N content, total N accumulation was also affected by seedling dry weight.

To elucidate the influence of different NH_4_^+^/NO_3_^–^ ratios on the distribution of N forms, we analyzed the content of NO_3_^–^, NH_4_^+^, free amino acids, and soluble protein in different tissues of flowering Chinese cabbage.

**FIGURE 3:**
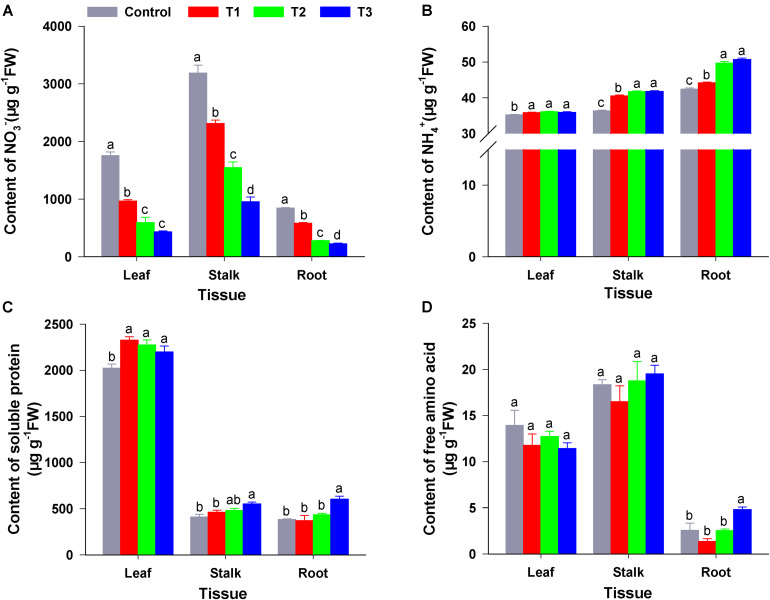
The content of N forms in the roots, stems, and leaves of flowering Chinese cabbage under different NH_4_^+^/NO_3_^–^ ratios. NO_3_^–^ content **(A)**; NH_4_^+^ content **(B)**; soluble protein content **(C)**; and free amino acid content **(D)**. Control = 0/100, T1 = 10/90, T2 = 25/75, T3 = 50/50. FW, fresh weight. The data represent mean ± SE (*n* = 3). Different letters indicate significant differences at *P* < 0.05.

As shown in Figure 3A, compared with the control (sole NO_3_^–^), the three treatments (T1–T3) significantly reduced the NO_3_^–^ content of roots, stems, and leaves of flowering Chinese cabbage seedlings; this was particularly visible in T3. In T3, the NO_3_^–^ content of roots, stems, and leaves was only 26.60, 29.88, and 24.76% that of the control, respectively; moreover, there were significant differences in NO_3_^–^ contents among the T1, T2, and T3. Similarly, compared to control, T1–T3 significantly reduced the content of NO_2_^–^ in different tissues; this was particularly visible in T3, in which the NO_2_^–^ content in roots, stems, and leaves was 36.11, 37.04, and 46.00% that of control, respectively (Supplementary Figure 1).

Compared with NH_4_^+^ content of control plants, NH_4_^+^ content in the roots, stalks, and leaves of plants in the three treatments were significantly increased. Moreover, NH_4_^+^ content increased with the increase in NH_4_^+^ concentration of the nutrient solution. NH_4_^+^ contents of plant roots, stalks, and leaves in T3 were 1.02, 1.15, and 1.20 times higher than those in the control (Figure 3B). The root amino acid content in T3 was significantly higher than that in the control and the other treatments; however, there was no significant difference between amino acid content in leaves and stalks among four treatments (Figure 3C). Compared to that of the control, soluble protein content in the leaves of flowering Chinese cabbage was significantly increased in T1–T3, whereas the soluble protein content of stalks and roots was significantly increased by T3 (1.34- and 1.56-fold higher than that in the stalks and roots of control plants). Furthermore, the soluble protein content of flowering Chinese cabbage leaves was much higher than that of stalks and roots (Figure 3D).

### Dynamic Changes in the Nutrient Solution Composition in Response to Different NH_4_^+^/NO_3_^–^ Ratios

To elucidate the effect of different NH_4_^+^/NO_3_^–^ ratios on flowering Chinese cabbage, we carried out dynamic monitoring of physicochemical properties of nutrient solution and N content of plants during the experimental period.

**FIGURE 4:**
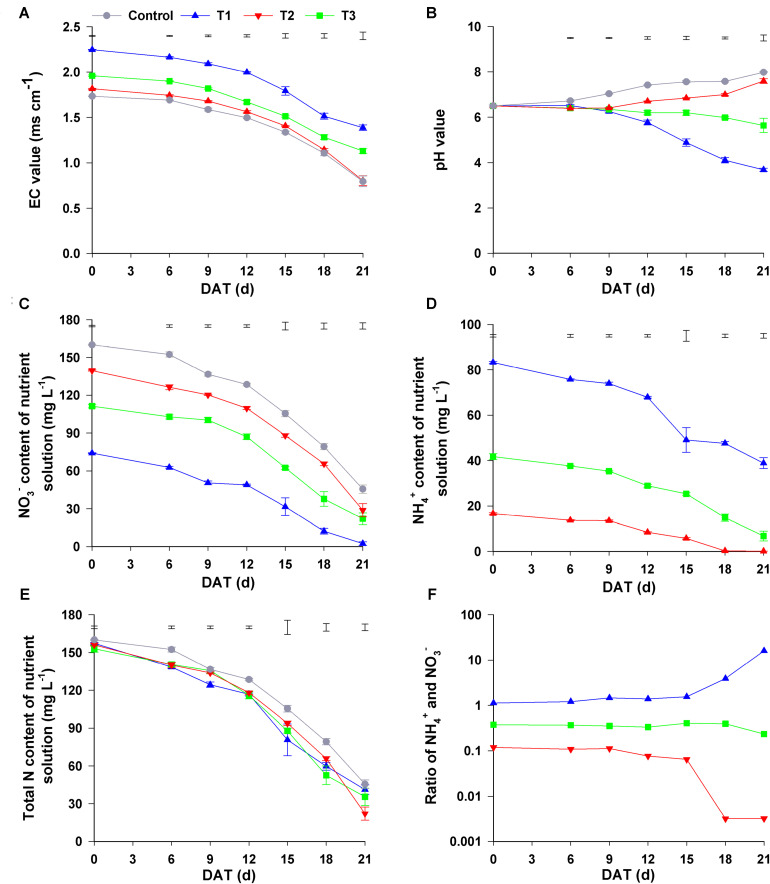
Changes in the nutrient solution composition under different NH_4_^+^/NO_3_^–^ ratios. **(A)** EC value; **(B)** pH value; **(C)** NO_3_^–^ content; **(D)** NH_4_^+^ content; **(E)** total N content; **(F)** ratio of NH_4_^+^ and NO_3_^–^. Control = 0/100, T1 = 10/90, T2 = 25/75, T3 = 50/50. DAT, days after treatment. The data represent mean ± SE (*n* = 3). The vertical ruler in **(A–E)** represents the LSD_0_._05_ of the average values among the treatments.

With the increase in NH_4_^+^ concentration of nutrient solutions, their initial EC value started to differ among the four treatments (1.70, 1.78, 1.91, and 2.21 mS cm^–1^ in control, T1, T2, and T3, respectively). This may be due to the increase in the number of ions in the nutrient solution and the interaction between these ions. During the experimental period, the EC value of all nutrient solutions gradually decreased, and the final EC values in control, T1, T2, and T3 were 0.44, 0.53, 0.71, and 1.26 mS cm^–1^, respectively (Figure 4A).

Changes in the pH value of the nutrient solutions were completely from the changes in EC value. During the experimental period, the pH values of the nutrient solutions in all four treatments significantly changed. Though the initial pH value of all nutrient solutions was 6.5, the pH values of the control solutions increased gradually until the end of experiment, and the final pH value was 8.14. In T1, the pH value of the nutrient solution decreased slowly (at 0–9 days after the start of the treatment), after which it quickly increased and continued to increase until the end of experiment; its final pH value was 7.86. In contrast, the pH value in T3 declined gradually at 0–9 days, after which it quickly decreased and was only 3.68 at 21 days after the start of the treatment. However, the change in the pH value in T2 nutrient solution was completely different from that in the other three treatments because pH was maintained in the range of 5.64–6.50 throughout the experimental period (Figure 4B). Consequently, the changes in the physicochemical properties of these nutrient solutions were distinctly different among the four treatments with different NH_4_^+^/NO_3_^–^ ratios during the experiment.

As shown in Figure 4C, the initial NO_3_^–^ content in the control, T1, T2, and T3 was 160.08, 139.55, 111.33, and 74.09 mg L^–1^, respectively, decreasing gradually with the decrease in NH_4_^+^/NO_3_^–^ ratio. During the plant growth period, the content of NO_3_^–^ in all nutrient solutions gradually decreased. The final concentration of NO_3_^–^ in the control, T1, T2, and T3 treatments was 45.56, 30.13, 18.79, and 2.43 mg L^–1^, respectively, the reduction of NO_3_^–^ with control, T1, T2, and T3 was 114.52, 109.42, 92.54, and 71.75 mg L^–1^, respectively. Similarly, NH_4_^+^ content of the nutrient solutions in T1–T3 decreased gradually as time progressed (Figure 4D). At harvest, the concentration of NH_4_^+^ in T1, T2, and T3 was 0.07, 6.73, and 38.85 mg L^–1^, with the decrease in NH_4_^+^ being 16.54, 35.00 and 44.41 mg L^–1^, respectively, compared to that at the beginning of the experiment. The total N content gradually decreased during the experimental period, and the greatest reduction in total N content was observed in T2 (the final content at harvest was 129.75 mg L^–1^), and the smallest was observed in control (the final content at harvest was 114.52 mg L^–1^) (Figure 4E).

To investigate the change of NO_3_^–^ and NH_4_^+^ contents in different treatments, we analyzed the ratio of NH_4_^+^ to NO_3_^–^ in the four treatments. In T1 and T3, during the first 15 d, the NH_4_^+^/NO_3_^–^ ratio was maintained at 0.10 and 1.10, respectively, after which this ratio in T1 decreased sharply to a stable level of 0.03 at 21 days, whereas in T3, it gradually increased to 16.02 at 21 days. Unlike that in the other three treatments, NH_4_^+^/NO_3_^–^ ratio in T2 changed little during the experimental period, staying in the range of 0.23–0.41 (Figure 4F). However, no significant change was observed in the contents and the ratio of NH_4_^+^/NO_3_^–^ in the nutrient solution without seedlings during the experimental period (Supplementary Figure 2). Therefore, the changes in nutrient solution composition may be related to the cultivation of flowering Chinese cabbage.

### N Loss and N Use Efficiency of Flowering Chinese Cabbage Plants in Response to Different NH_4_^+^/NO_3_^–^ Ratios

To evaluate the N utilization of flowering Chinese cabbage plants in response to different NH_4_^+^/NO_3_^–^ ratios, we further analyzed the N loss, N productivity, N residence time, and NUE. We observed that a certain proportion of N loss occurred when flowering Chinese cabbage were grown under hydroponic conditions. Among the four treatments, T3 which had the highest NH_4_^+^/NO_3_^–^ ratio in the nutrient solution (50/50), had significantly higher N loss than the other three treatments, among which there was no significant difference. Regarding the rate of N loss, it was lower in T2 than in the control, but it was significantly higher in T1 and T3 than in the control owing to the shorter or longer residence time of N remaining in the roots (Table 2). The rate of N loss was the highest in T3; it was 1. 44-, 1. 37-, and 1.51-fold that of the control, T1, and T2, respectively. Compared to that in control, N productivity was 1.18, 1.19, and 1.12 times higher in T1, T2, and T3, respectively. Owing to the differences in the N loss rate and N productivity, NUE was significantly different among the four treatments. NUE was the highest in T2, being 1. 22-, 1. 18-, and 1.74-fold that of the control, T1, and T3, respectively; it was the lowest in T3, being only 70.55% that of the control. Therefore, compared to the N loss in the control treatment (sole NO_3_^–^), the appropriate proportion of NH_4_^+^/NO_3_^–^ could reduce the rate of N loss, prolong the N residence time, and improve N productivity and NUE, as was the case with T2 (NH_4_^+^/NO_3_^–^ = 25/75); nevertheless, a higher ratio of NH_4_^+^/NO_3_^–^ could have the opposite effect.

**TABLE 2:** Rate of N loss, N residence time, N productivity, and N use efficiency in nutrient solutions with different NH_4_^+^/NO_3_^–^ ratios during the growth period of flowering Chinese cabbage.

Treatment	N loss (mg plant^–1^)	Rate of N loss (%)	N residence time (d)	N productivity (mg mg^–1^ d^–1^)	N use efficiency (mg mg^–^^1^)
Control	52.59 ± 5.31b	17.92 ± 1.81b	18.59 ± 2.26a	5.36 ± 0.21b	98.78 ± 4.64b
T1	54.02 ± 2.81b	18.87 ± 0.98*ab*	16.01 ± 1.18*ab*	6.34 ± 0.21a	101.53 ± 4.55b
T2	47.87 ± 4.69b	17.06 ± 1.67b	19.05 ± 2.71a	6.39 ± 0.24a	120.53 ± 7.26a
T3	74.43 ± 4.64a	25.80 ± 1.61a	11.66 ± 0.93b	5.98 ± 0.22*ab*	69.39 ± 1.81c

*Control = 0/100, T1 = 10/90, T2 = 25/75, T3 = 50/50. The data represent mean ± SE (n = 3). Different letters indicate significant differences at P < 0.05.*

### Xylem Exudate of Flowering Chinese Cabbage Plants in Response to Different NH_4_^+^/NO_3_^–^ Ratios

**FIGURE 5:**
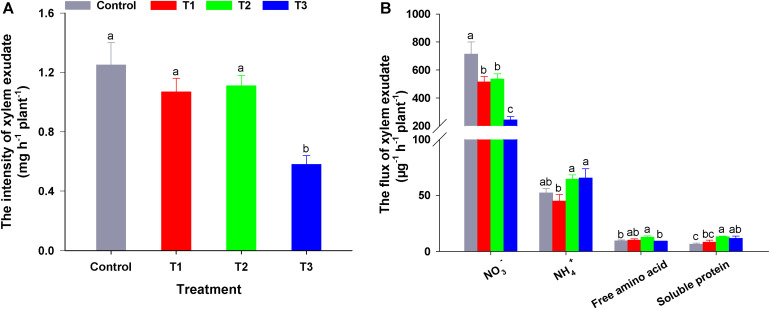
The intensity **(A)** and flux **(B)** of xylem exudate in flowering Chinese cabbage in response to different NH_4_^+^/NO_3_^–^ ratios. Control = 0/100, T1 = 10/90, T2 = 25/75, T3 = 50/50. The data represent mean ± SE (*n* = 3). Different letters indicate significant differences at *P* < 0.05.

To elucidate the influence of different NH_4_^+^/NO_3_^–^ ratios on N allocation, we analyzed the xylem exudate composition of flowering Chinese cabbage seedlings. To ensure that we could obtain exudates in all treatments, we constantly adjusted the pH values of the nutrient solutions to approximately 6.5 during the experiment. There was no significant difference in the root activity measured by TTC among the four treatments (Supplementary Figure 4). However, the exudation intensity in T3 was significantly lower than that in the other three treatments (i.e., 46.90, 55.18, and 53.60% lower than that of control, T1, and T2, respectively) (Figure 5).

Compared with NO_3_^–^ flux in the control, the addition of NH_4_^+^ to the nutrient solution significantly reduced the flux of NO_3_^–^ in the exudates, and this effect was more pronounced with the increase in NH_4_^+^ concentration, (in T3, NO_3_^–^ flux in the exudates was only 34.11% that of the control). Regarding the NH_4_^+^ flux in the exudates, compared to the control, T1 decreased the NH_4_^+^ flux, which was lower than that of T2 or T3. Even though the NH_4_^+^ concentration of T2 nutrient solution was half that of T3 nutrient solution, there was no significant difference in NH_4_^+^ flux between these two treatments (Figure 5B). Compared with the control, the flux of soluble proteins in the exudates of flowering Chinese cabbage was up-regulated by three NH_4_^+^/NO_3_^–^ ratios treatments, and the soluble protein flux was the highest in T2, being nearly twice the control. Free amino acid flux was similar to that of soluble proteins. Among the exudation fluxes of 4 N-forms, NO_3_^–^ flux was the greatest. The proportion of NO_3_^–^ in total N was 91.27, 89.05, 85.55, and 73.76% in control, T1, T2, and T3, respectively. This indicated that NO_3_^–^ is the main form of N transported from the roots to the shoots in flowering Chinese cabbage. The contents of different N forms in the exudates of flowering Chinese cabbage were similar to the changes of N flux in all treatments (Supplementary Table 2). In summary, we confirmed that different NH_4_^+^/NO_3_^–^ ratios had significantly different effects on plant N flux.

### Expression of Plasma Membrane H^+^-ATPase Genes (*HAs*) in Flowering Chinese Cabbage Plants in Response to Different NH_4_^+^/NO_3_^–^

The pH value of nutrient solutions changed significantly in response to different NH_4_^+^/NO_3_^–^ ratios (Figure 4B). PM H^+^-ATPase plays an important role in intracellular pH homeostasis and ensures normal life activities (Duby and Boutry, 2009). Thus, we analyzed the characteristics of *HA* genes encoding PM H^+^-ATPase proteins.

**FIGURE 6:**
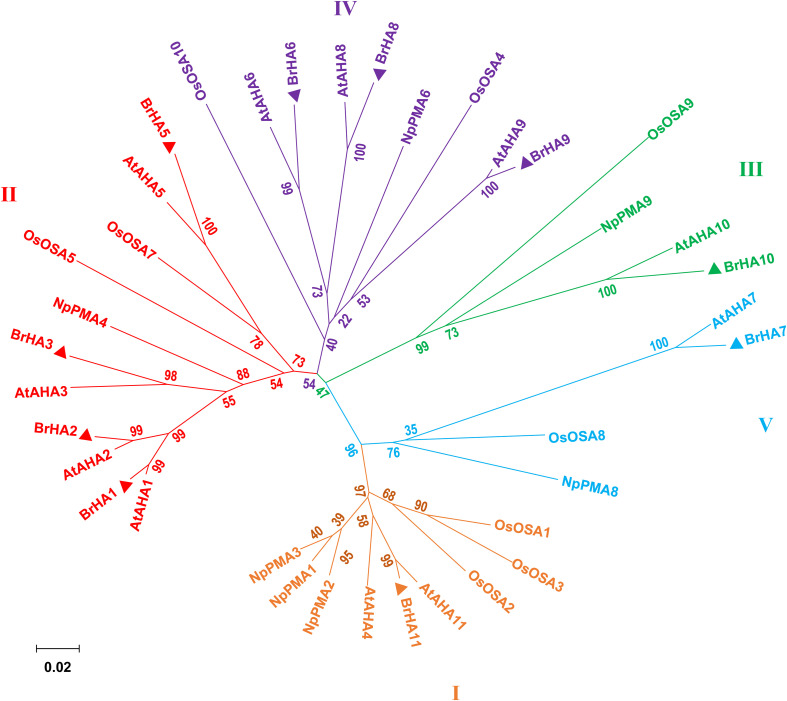
Phylogenetic tree showing the relationships among the plasma membrane H^+^-ATPases from *Brassica rapa* (Br), *Arabidopsis thaliana* (At), *Oryza sativa* (Os), and *Nicotiana plumbaginifolia* (Np). Phylogenetic trees were built using the MEGA6.0 (for protein ID codes, see Supplementary Table 4). Bootstrap values from 1,000 replicates were used to estimate the confidence limits of the nodes. The scale bar represents a 0.02 estimated amino acid substitutions per residue. Triangles represent the PM H^+^-ATPase protein from *Brassica rapa*.

As described by Arango et al. (2003), the putative PM H^+^-ATPase is distributed into five subfamilies in the phylogenetic tree constructed from *Arabidopsis thaliana* (At), *Oryza sativa* (Os), *Nicotiana plumbaginifolia* (Np), and *Brassica rapa* (Br). Seven out of ten putative proteins were clustered in two of the five subfamilies: LOC103855020 (BrHA11), LOC103835963 (BrHA10), and LOC103841882 (BrHA7) were clustered in subfamily I, III, and V, respectively; LOC103828351 (BrHA1), LOC103834686 (BrHA2), LOC103845176 (BrHA3), and LOC10 3842249 (BrAHA5) were clustered in subfamily II; and LOC103842249 (BrHA6), LOC103853056 (BrHA8), and LOC103830447 (BrHA9) were clustered in subfamily IV (Figure 6).

**FIGURE 7:**
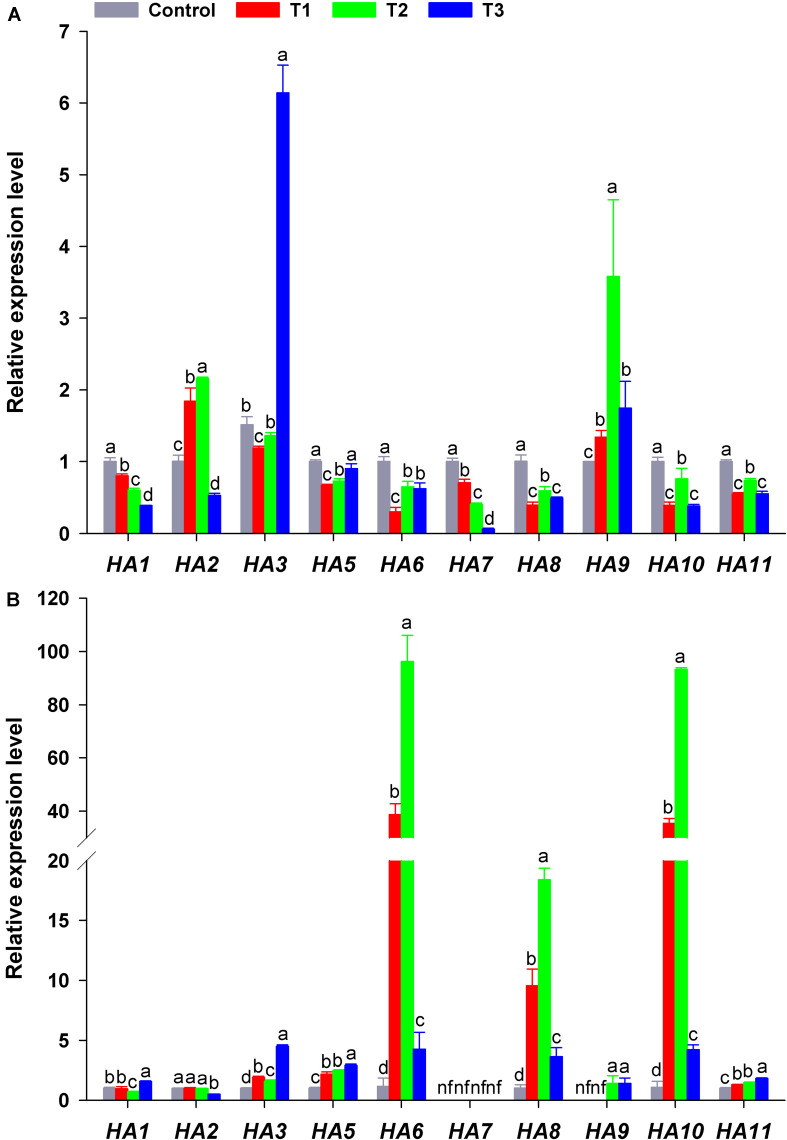
Expression level of plasma membrane H^+^-ATPase genes (*HAs*) in the roots **(A)** and leaves **(B)** of flowering Chinese cabbage plants in response to different NH_4_^+^/NO_3_^–^ ratios during the 21 days of the experiment. Control = 0/100, T1 = 10/90, T2 = 25/75, T3 = 50/50. The data represent mean ± SE (*n* = 3). Different letters indicate significant differences at *P* < 0.05. nf represents not detected.

*HA* genes in flowering Chinese cabbage had different response to different NH_4_^+^/NO_3_^–^ ratios. The expression of *HA1* or *HA7* in plant roots decreased significantly with the increase in NH_4_^+^/NO_3_^–^ ratios, especially in T3 (their expression was only 39 and 6% that of the control, respectively) (Figure 7A). In contrast, *HA3* transcription in T3 was higher than that in the other treatments, being 4. 06-, 5. 17-, and 4.51-fold that of the control, T1, and T2, respectively. The transcription of *HA2* and *HA9* was higher in T2 than in the other treatments. As for the expression of *HA5*, the expression in the control and T3 was higher than that in T1 and T2, whereas the expression of *HA6*, *HA8*, *HA10*, and *HA11* was similar in response to different NH_4_^+^/NO_3_^–^ ratios, even though it was higher in the control and T2 than in T1 and T3 (Figure 7A).

In plant leaves, the expression of *HA3*, *HA5*, *HA9*, and *HA11* significantly increased with the increase in NH_4_^+^/NO_3_^–^ ratios, especially in T3 (NH_4_^+^/NO_3_^–^ = 50/50). However, the expression of *HA6*, *HA8*, and *HA10* increased at first and then decreased with the increase in NH_4_^+^/NO_3_^–^ ratios, especially in T2 (NH_4_^+^/NO_3_^–^ = 25/75), where their expression was the highest, being 83.64, 17.94, and 85.35 times that of the control. The transcription of *HA1* and *HA2* in plant leaves was little affected by different NH_4_^+^/NO_3_^–^ ratios, and the expression of *HA7* in plant leaves was not detected in any treatment (Figure 6). Overall, the expression of *HAs* was significantly affected by different NH_4_^+^/NO_3_^–^ ratios.

## Discussion

### Response of Flowering Chinese Cabbage Plants Growth to Different NH_4_^+^/NO_3_^–^ Ratios

As the main forms of inorganic N, both NH_4_^+^ and NO_3_^–^ can be taken up by plants; however, they play different roles in photosynthesis (Konnerup and Brix, 2010), the absorption of minerals (Zou et al., 2001; Zeng et al., 2012) and water (Ding et al., 2018), and other physiological and biochemical processes. Previous studies have reported the effects of different NH_4_^+^/NO_3_^–^ ratios on the biomass of several plant species (Tabatabaei et al., 2008; Santos et al., 2013; Hu et al., 2015; Song et al., 2017). In the present study, the results showed that the appropriate NH_4_^+^ and NO_3_^–^ ratios (i.e., NH_4_^+^/NO_3_^–^ = 10/90 and 25/75) were beneficial to the growth of flowering Chinese cabbage and resulted in higher fresh weight and economical yield, especially for the treatment of NH_4_^+^/NO_3_^–^ = 25/75. Santos et al. (2013) reported that supplying N in a nutrient solution in the form of NO_3_^–^ or NH_4_^+^ exceeding 70% or 50%, respectively, may decrease the yield in *Panicum maximum*. However, in the present study, the high NH_4_^+^/NO_3_^–^ ratio (50/50) significantly accelerated the growth of seedlings at the early stage (0–12 days), and it inhibited it at the late stage (15–21 days); during this period, plant growth rate was 2.45 mg d^–1^ plant^–1^, which was only 36.57% of plant growth in control with sole NO_3_^–^ (Figure 1 and Table 1). This showed that the effects of different forms of N on plant growth could be also closely related to the duration of treatment, and that the highest concentration of NH_4_^+^ used in our study would not cause ammonium toxicity when applied for only a short time. In our experiment involving different NH_4_^+^/NO_3_^–^ ratios, other cations or anions present in the nutrient solution may be involved with supplying together with the N source, and their concentrations were always affected, as indicated by different original EC value of the nutrient solution among four treatments (Figure 4A). In this study, 2.25, 5.60, and 11.23 mmol L^–1^ Cl^–^ was added to the T1, T2, and T3 nutrient solution, respectively, and these concentrations were below the threshold of osmotic salt stress, which is approximately 40 mmol L^–1^ NaCl for most plants (Munns and Tester, 2008). Therefore, the amount of Cl^–^ in nutrient solution in present study was insufficient to cause salt stress. Furthermore, we analyzed the content and ratio of NH_4_^+^ and NO_3_^–^ in the nutrient solution. We found that the NH_4_^+^/NO_3_^–^ ratio in the treatment with NH_4_^+^/NO_3_^–^ = 50/50 remained at 1.10 at the early stage and increased rapidly at the late stage of plant growth, whereas that in the treatment with NH_4_^+^/NO_3_^–^ = 27/75 remained unchanged throughout the entire growth period (Figure 4F). Thus, we speculated that the appropriate ratios of NH_4_^+^ and NO_3_^–^ in the rhizosphere environment could be beneficial for the growth of flowering Chinese cabbage.

### Response of Plant Absorption and Accumulation of Nutrient Elements to Different NH_4_^+^/NO_3_^–^ Ratios

Dry matter and nutrient accumulation are necessary for organ differentiation and yield formation, and the absorption of nutrients is the basis for dry matter formation and accumulation. Song et al. (2017) reported that different ratios of NH_4_^+^/NO_3_^–^ had different influence on the content and accumulation of N in plants. Our study showed that the total N content of flowering Chinese cabbage was significantly increased by the three treatments (T1–T3) at 0–18 days after treatment compared with that of the control treatment with sole NO_3_^–^ (Figure 2A). Regarding the accumulation of total N, in T2 (NH_4_^+^/NO_3_^–^ = 25/75), it was significantly increased in contrast to that in the other three treatments over the entire growth period of flowering Chinese cabbage owing to the increase in plant biomass (Figure 2B). This finding was similar to previous findings (Song et al., 2012; Yin et al., 2020).

Moreover, the content of different N compounds in the tissues of flowering Chinese cabbage was significantly affected by different ratios of NH_4_^+^/NO_3_^–^. At harvest, the NO_3_^–^ and NO_2_^–^ content in leaves, stalks, and roots significantly declined as the NO_3_^–^ content of the nutrient solution decreased, whereas NH_4_^+^ content exhibited the opposite trend, increasing as the NH_4_^+^ content in the nutrient solution increased (Figures 3A,B and Supplementary Figure 1). The changes in free amino acid and soluble protein contents were similar to the changes in NH_4_^+^ content (Figures 3B–D). This was in accordance with the results reported by Arnold et al. (2015), and we confirmed the hypothesis that higher costs of NO_3_^–^ assimilation, which is used for amino acid synthesis, result in overall lower amino acid synthesis rates, compared with that when a mix of NH_4_^+^ and NO_3_^–^ is assimilated by plants. Through the analysis of xylem sap, we also confirmed that the flux and content of NO_3_^–^, NH_4_^+^, soluble protein, and free amino acid in the exudates showed a similar tendency in response to different NH_4_^+^/NO_3_^–^ ratios (Figure 5B and Supplementary Table 2).

The absorption of NO_3_^–^ or NH_4_^+^ by plants results in rhizosphere alkalization or acidification, respectively, which affects not only N absorption but also the absorption of other mineral nutrients (Sarasketa et al., 2016). In the present study, the pH value of the nutrient solution gradually declined in T3 (NH_4_^+^/NO_3_^–^ = 50/50), gradually increased in the control (sole NO_3_^–^) and T1 (NH_4_^+^/NO_3_^–^ 10/90), and slightly declined in T2 (NH_4_^+^/NO_3_^–^ = 25/75; pH = 5.64 at harvest) (Figure 4B). Response of plant growth and nutrient absorption to the addition of different N forms may vary with changes in external pH, which may in turn modify pH value (Gerendas and Schurr, 1999).

In plants, the transport and assimilation of NO_3_^–^ and NH_4_^+^ can dominate cellular pH homeostasis, which in turn affects the availability and utilization of other nutrients (Feng et al., 2020). The absorption of K and P was significantly changed by NH_4_^+^ and NO_3_^–^ (Supplementary Figures 2A–D). In the present study, the three treatments significantly increased the content and accumulation of P compared with those in the control treatment (Supplementary Figures 2A,B), whereas the high ratio of NH_4_^+^/NO_3_^–^ significantly inhibited the absorption of K (Supplementary Figures 2C,D). The content and flux of P and K in the exudates of flowering Chinese cabbage showed a similar tendency (Supplementary Table 3). Previous studies have shown that a low NO_3_^–^/NH_4_^+^ ratio inhibits plant growth and changes ion balance, as well as that the absorption of inorganic cations (K^+^, Ca^2+^, and Mg^2+^) is reduced under high NH_4_^+^ conditions (Britto and Kronzucker, 2002). Zeng et al. (2012) reported that PM H^+^-ATPase is involved in the stimulated uptake of P by rice roots supplemented with NH_4_^+^. Thus, the changes in rhizosphere pH caused the absorption of NH_4_^+^ or NO_3_^–^ had important influence on the absorption of other nutrient elements, which may have been caused by the interaction between ions.

### Response of NUE and N Loss to Different NH_4_^+^/NO_3_^–^ Ratios

Previous reports have indicated that nutrient use efficiency may be expressed in different ways (Xu et al., 2012). In the present study, nutrient use efficiency was described as reported by Berendse and Aerts (1987), where nitrogen use efficiency is divided into N productivity and average N retention time, which can reflect a rapid growth strategy of plants and a nutrient retention strategy, respectively. We observed that compared with control and other treatments, treatments with moderate ratios of NH_4_^+^ and NO_3_^–^ (NH_4_^+^/NO_3_^–^ = 25/75) improved plant N use efficiency by increasing N productivity and extending the average N retention time, whereas the high ratio of NH_4_^+^ and NO_3_^–^ (NH_4_^+^/NO_3_^–^ = 50/50) significantly reduced plant NUE by shortening the average nitrogen retention time (Table 2). Thus, N loss in the treatment with high ratio of NH_4_^+^ and NO_3_^–^ was significantly higher than that in the control treatment with sole NO_3_^–^.

The major pathways for N losses include leaching to surface- and groundwater, soil erosion, NH_3_ volatilization, N_2_O and NO_*x*_ fluxes to the atmosphere, and N_2_ denitrification (Xu et al., 2012). In the present study, the contents of NH_4_^+^, NO_3_^–^, and total N in the nutrient solution without plant culture were almost constant during the growth period (Supplementary Figure 3). Thus, it was evident that N loss is low in a closed hydroponics system owing to reduced soil leaching and surface runoff. It could be inferred that the loss of N may have been related to the N metabolism of plants or caused by the change in nutrient solution composition during plant cultivation. Among the N loss pathways, NH_3_ volatilization is a major pathway for N loss, and up to 23% of applied N can be lost via NH_3_ volatilization (Ju and Zhang, 2017), which can be a consequence of photorespiration, NO_3_^–^ reduction, amino acid catabolism, and phenylpropanoid metabolism (Bittsánszky et al., 2015). The unbalanced accumulation and transformation of active N sources (i.e., NH_4_^+^, NO_3_^–^, and NO_2_^–^) in plant leaves is the fundamental source of N volatilization loss in plants (Xu et al., 2012). In the present study, we observed that the ratio of NH_4_^+^ to NO_3_^–^ in nutrient solution in T3 (NH_4_^+^/NO_3_^–^ = 50/50) was up to 16.02 in the late stage of the experiment, whereas in T2 (NH_4_^+^/NO_3_^–^ = 25/75), this ratio was maintained in the range of 0.23–0.41 (Figure 4D), which might have contributed to balanced N absorption. In contrast, nutrient solutions with higher NH_4_^+^/NO_3_^–^ ratio may cause insufficient assimilation of NH_4_^+^ after its absorption or protein degradation owing to NH_4_^+^ toxicity, which can increase the loss of N. Therefore, the appropriate ratio of NH_4_^+^ to NO_3_^–^ (i.e., NH_4_^+^/NO_3_^–^ = 25/75 for flowering Chinese cabbage) can decrease N loss, improve NUE, and optimize crop performance.

### Xylem Exudate Analysis Supported the Results of Nutrient Absorption and Assimilation Analysis Under Different NH_4_^+^/NO_3_^–^ Ratios

In plants, nutrients are mainly transported from roots to shoots through the xylem, and the intensity and composition of exudates can reflect plant nutrition status (Buhtz et al., 2004). In the present study, medium and high NH_4_^+^/NO_3_^–^ ratios significantly reduced the NO_3_^–^ content and flux in the xylem exudates compared with that in control treatment with sole NO_3_^–^, while significantly increasing NH_4_^+^ content and flux (Figure 5 and Supplementary Table 2). This result was similar to the results in rice (Kronzucker et al., 1999) and *Populus popularis* (Luo et al., 2013). Besides the difference in energy source between sole NO_3_^–^ and the mixture of NH_4_^+^ and NO_3_^–^ (Hachiya et al., 2012), Arnold et al. (2015) reported that NH_4_^+^ and NO_3_^–^ might interact with each other when they are both present in the nutrient solution. Our previous results obtained using a scanning ion-selective electrode technique in flowering Chinese cabbage showed that the absorption of NH_4_^+^ may be accelerated by the addition of NO_3_^–^ into the nutrient solution, whereas the addition of NO_3_^–^ had the opposite effect (Zhu et al., 2020).

NH_4_^+^ content in plant exudates can be a consequence of direct NH_4_^+^ uptake, but also a consequence of the decrease in NO_3_^–^, amino acid deamination, protein degradation, and photorespiration (Sarasketa et al., 2014). However, we observed that there was a slight difference in the NH_4_^+^ flux of the exudates between T3 (NH_4_^+^/NO_3_^–^ = 50/50) and T2 (25/75), although the NH_4_^+^ content of T3 nutrient solution in was twice of T2 (Table 2). To avoid excessive NH_4_^+^ absorption which would result in ammonium toxicity, plants adopt different strategies, such as the regulation of AMT activity (Bittsánszky et al., 2015) and increase in NH_4_^+^ assimilation (Setien et al., 2013). In the present study, the flux or content of soluble protein or free amino acid in the xylem exudates increased with the increase in NH_4_^+^/NO_3_^–^ ratio (Figure 5B and Supplementary Table 2); this was similar to the results reported by Yin et al. (2020). However, the flux of soluble protein or free amino acid under T3 (NH_4_^+^/NO_3_^–^ = 50/50) was significantly decreased compared to that under T2 (NH_4_^+^/NO_3_^–^ = 25/75) (Figure 5B). Furthermore, during the later period, the seedlings in the treatment with NH_4_^+^/NO_3_^–^ = 50/50 showed symptoms of ammonia toxicity, including leaf etiolation, root reddening, and a lower growth rate and less biomass than the seedlings in the other treatments (Figure 1 and Table 1). This implied that the seedlings of flowering Chinese cabbage under T3 might have experienced ammonia toxicity. Esteban et al. (2016) showed that complementation of NH_4_^+^ with NO_3_^–^ at low doses, which has long been practiced in agriculture, can counterbalance the toxicity of NH_4_^+^ in laboratory conditions. Furthermore, NO_3_^–^ may accelerate the transport of NH_4_^+^ or the assimilation product from roots to shoots (Hachiya et al., 2012; Hachiya and Sakakibara, 2017). Consequently, the absorption and transport of NH_4_^+^, NO_3_^–^, and their assimilation products may be affected by different NH_4_^+^/NO_3_^–^ ratios. Therefore, applying appropriate ratios of NH_4_^+^/NO_3_^–^ is one of the important ways to improve plant NUE (Rouphael et al., 2018).

### Response of Plasma Membrane H^+^-ATPase to Different NH_4_^+^/NO_3_^–^ Ratios

Generally, the absorption of NO_3_^–^ or NH_4_^+^ by plants results in alkalization or acidification of the rhizosphere (Sarasketa et al., 2016), this is related to the H^+^ electrochemical gradient, which is controlled by H^+^-ATPase in the PM (Haruta et al., 2018). In the present study, the pH values of the nutrient solution with full NO_3_^–^ and that with a low ratio of NH_4_^+^/NO_3_^–^ (10/90) were significantly increased, whereas the pH of the solution with a high ratio NH_4_^+^/NO_3_^–^ (50:50) was sharply reduced, being only 3.68 at the time of harvest; the pH of NH_4_^+^/NO_3_^–^ 25/75 solution was maintained in the range of 5.64–6.50 (Figure 4B). Zhu et al. (2009) have reported that the biomass of rice seedlings supplied with NH_4_^+^ at pH 6.5 is significantly higher than those given NO_3_^–^, whereas there is no difference between the two N forms at pH 3.0, in which PM H^+^-ATPase plays an important role. PM H^+^-ATPase pumps create the electrochemical H^+^ gradients which energize most transport processes in and out of plant cells through channel proteins and secondary active carriers (Hoffmann et al., 2019).

It is generally believed that the mechanism through which the supply of NH_4_^+^ acidifies the rhizosphere is related to active PM H^+^-ATPase pumping H^+^ from intracellular into extracellular space, to maintain H^+^ homeostasis in root cells (Palmgren and Harper, 1999; Zhang et al., 2018). The differences in PM H^+^-ATPase activity between NH_4_^+^-supplied and NO_3_^–^-supplied roots could be related to the discrepancy in H^+^ generation during the assimilation of NH_4_^+^ and NO_3_^–^ (Zhang et al., 2018), H^+^-ATPase activity shows a narrow pH optimum at pH 6.0 (Zhu et al., 2009). Furthermore, PM H^+^-ATPase is involved in the ammonium-nutrition response of plant roots (Zhu et al., 2009; Zhang et al., 2017), the activity of H^+^-pumping, H^+^-ATPase, and pH gradient across PM were significantly related the pH value of nutrient solution, rather than the N forms (Zhu et al., 2009). Zhu et al. (2009) reported that a high expression of various PM H^+^-ATPase genes (*OSA1*, *OSA3*, *OSA7*, *OSA8*, and *OSA9*) is responsible for the adaptation of rice roots to low pH, either under sole NH_4_^+^ or sole NO_3_^–^. Similarly, PM H^+^-ATPase also participates in NO_3_^–^ uptake in plants (Sperandio et al., 2011; Pii et al., 2014). In rice, *OSA2*, *OSA5*, *OSA7*, and *OSA8* are induced by NO_3_^–^ (Sperandio et al., 2011), which consequently increases N accumulation and rice growth (Sperandio et al., 2014); in winter wheat, *TaHA1* also participates in this process (Jiang et al., 2017); the expression and activity of *VvHA2* and *VvHA4* are induced by NO_3_^–^ in grapevine (Pii et al., 2014).

To elucidate how different NH_4_^+^/NO_3_^–^ ratios influence N absorption and assimilation, we further analyzed the phylogenetic tree and transcription level of PM H^+^-ATPase gene (*HAs*) in flowering Chinese cabbage. A previous phylogenetic analysis based on the homology of sequences showed that 11 genes encoding HA homologs were present in *Brassica rapa* genome and were clustered into five clades, which was similar to the results obtained for *Arabidopsis* and *Nicotiana tabacum* (Arango et al., 2003). The predicted proteins encoded by *BrHA1*, *BrHA2*, *BrHA3*, and *BrHA5* are clustered in clade II; those encoded by *BrHA6*, *BrHA8*, and *BrHA9* are clustered in clade IV; and those encoded by *BrHA7*, *BrHA10*, and *BrHA11* are clustered in clades V, III, and I (Figure 6). Most members of subfamilies I and II are usually expressed in all tissues, whereas the expression of the members of other subfamilies is dependent on the tissue or environmental factors (Arango et al., 2003). A similar tendency was observed in the present study: *BrHA1*, *BrHA2*, *BrHA3*, *BrHA5*, and *BrHA11* were expressed in roots and leaves, while *BrHA7* and *BrHA9* were either not expressed or expressed only in roots of flowering Chinese cabbage (Figures 7A,B).

Consistent with the results of previous studies (Zhu et al., 2009; Sperandio et al., 2011; Pii et al., 2014), the expression of *BrHAs* in flowering Chinese cabbage was obviously affected by different NH_4_^+^/NO_3_^–^ ratios. The expression of *BrHA1*, *BrHA5-8*, *BrHA10*, and *BrHA7* in roots and *BrHA2* in leaves was decreased by the addition of the mixture of NH_4_^+^ and NO_3_^–^, whereas the expression of *BrHA3* in roots and *BrHA1*, *BrHA3*, *BrHA5*, *BrHA11* in leaves was increased by high NH_4_^+^/NO_3_^–^ ratios. In contrast, the expression of *BrHA2* and *BrHA9* in roots and *BrHA6*, *BrHA8*, and *BrHA10* in leaves was significantly up-regulated by low NH_4_^+^/NO_3_^–^ ratios compared with that in sole NO_3_^–^ or high NH_4_^+^/NO_3_^–^ ratio treatments (Figures 7A,B). The results were similar to those described by Zhang et al. (2018) in barley, the expression of *HvHA3* and *HvHA9* was down-regulated by NO_3_^–^, but *HvHA7* expression was induced by NO_3_^–^. In *Arabidopsis*, AHA2 is important for the growth and development of roots regardless of N levels, and this correlation may be related to the control of pH homeostasis (Młodzińska et al., 2015); AHA7 mainly functions when apoplastic pH is above 6.0, and it can make up for the lack of AHA2 (Hoffmann et al., 2019). *OSA7* in rice is induced by NO_3_^–^ (Sperandio et al., 2014), which plays an important role in rice root growth and grain production without affecting N accumulation; this may highlight the importance of other PM H^+^-ATPase isoforms in N uptake (Sperandio et al., 2020). Expression studies with *HA3*, *HA6*, and *HA8-10* suggest that they participate in response to different forms of N (Figures 7A,B; Pii et al., 2014; Zhang et al., 2018), but again, genetic evidence for this essential function is lacking (Haruta et al., 2018). In the present study, the expression level of *HA7*, along with the expression level of *HA1* in roots, was positively correlated with the pH of nutrient solution, which decreased as the NH_4_^+^/NO_3_^–^ ratios increased (Figure 4B). Whether pH homeostasis is regulated by BrHA1/BrHA7 in flowering Chinese cabbage roots is worth investigating in future studies.

In addition to controlling the nutrient fluxes across the PM, PM H^+^-ATPase is involved in several other physiological processes, such as stomatal opening, phloem transport, and cell growth (Arango et al., 2003; Duby and Boutry, 2009). We observed that the stomata of flowering Chinese cabbage leaves in T3 (NH_4_^+^/NO_3_^–^ = 50/50) were closed, whereas in the other treatments, they were open (Supplementary Figure 5). This indicates that the appropriate NH_4_^+^/NO_3_^–^ ratios might help maintain normal stomata opening and contribute to gas exchange between plants and the atmosphere, thereby benefitting photosynthesis; on the contrary, higher NH_4_^+^/NO_3_^–^ ratios induce the closure of stomata, thus reducing water absorption, transpiration, and mineral absorption, and especially diminishing NH_4_^+^ uptake to avoid ammonia toxicity, the symptoms of which were observed during the later period. The activity of PM H^+^-ATPase is regulated by abscisic acid, ethylene, and other hormones (Chen et al., 2017). Kinoshita and Hayashi (2011) reported that blue light can induce the activation of H^+^-ATPase via phosphorylation of penultimate threonine (Thr) in the C-terminal H^+^-ATPase and trigger subsequent binding of the 14-3-3 protein to the phosphorylated H^+^-ATPase. The mechanism of H^+^-ATPase action in different N conditions should be investigated in the future.

In summary, supplying an appropriate ratio of NH_4_^+^/NO_3_^–^ (NH_4_^+^/NO_3_^–=^25/75) can improve N absorption and assimilation and promote the growth of flowering Chinese cabbage owing to the suitable pH value; on the contrary, the addition of excessive NH_4_^+^ may induce rhizosphere acidification and ammonia toxicity, resulting in growth inhibition. Our results provide valuable information regarding the influence of different NH_4_^+^/NO_3_^–^ ratios on plant growth and N uptake and utilization.

## Data Availability Statement

The original contributions presented in the study are included in the article/Supplementary Material, further inquiries can be directed to the corresponding authors.

## Author Contributions

SS and RC conceived and designed the research. BQ carried out the experiments. YZ analyzed the data and wrote the manuscript. YH helped to analyze the data and reviewed the manuscript. GS and HL reviewed and edited the manuscript. All authors contributed to the article and approved the submitted version.

## Conflict of Interest

The authors declare that the research was conducted in the absence of any commercial or financial relationships that could be construed as a potential conflict of interest.

## Supplementary Material

Supplementary Figure 1 NO_2_^–^ content in root, stem and leaf of flowering Chinese cabbage under different NH_4_^+^/NO_3_^–^ ratios.

Supplementary Figure 2 Effect of different NH_4_^+^/NO_3_^–^ ratios on the content and accumulation of total P and K in the growth period of flowering Chinese cabbage.

Supplementary Figure 3 The change of NO_3_^–^, NH_4_^+^ and total N content of the nutrient solution without seedlings under different NH_4_^+^/NO_3_^–^ ratios.

Supplementary Figure 4 The root activity of flowering Chinese cabbage under different NH_4_^+^/NO_3_^–^ ratios.

Supplementary Figure 5 Stomatal conductance of leaf in flowering Chinese cabbage in response to different NH_4_^+^/NO_3_^–^ ratios.

Supplementary Table 1 The primers were used in this paper.

Supplementary Table 2 The content of xylem exudate in flowering Chinese cabbage in response to different ratios of NH_4_^+^ and NO_3_^–^.

Supplementary Table 3 The content and flux of P and K in xylem exudate in flowering Chinese cabbage in response to different ratios of NH_4_^+^/NO_3_^–^.

Supplementary Table 4 The name and accession number of the H^+^-ATPase protein used for the generation of the phylogenetic tree.

## Abbreviations

AMT: ammonium transporter
GAPDH: glyceraldehyde-3-phosphate dehydrogenase
HA: H^+^-ATPase
N: nitrogen
NH_4_^+^: ammonium
NO_3_^–^: nitrate
NRT: nitrate transporter
PM: plasma membrane
qPCR: quantitative real-time polymerase chain reaction
TTC: 2, 3, 5-tetraphenyltetrazolium chloride.
