# Supplementary figures and images for: Appropriate NH4+/NO3– Ratio Triggers Plant Growth and Nutrient Uptake of Flowering Chinese Cabbage by Optimizing the pH Value of Nutrient Solution
Source: Front Plant Sci. 2021 Apr 30;12:656144. doi: 10.3389/fpls.2021.656144 (PMC8121088; doi:10.3389/fpls.2021.656144)

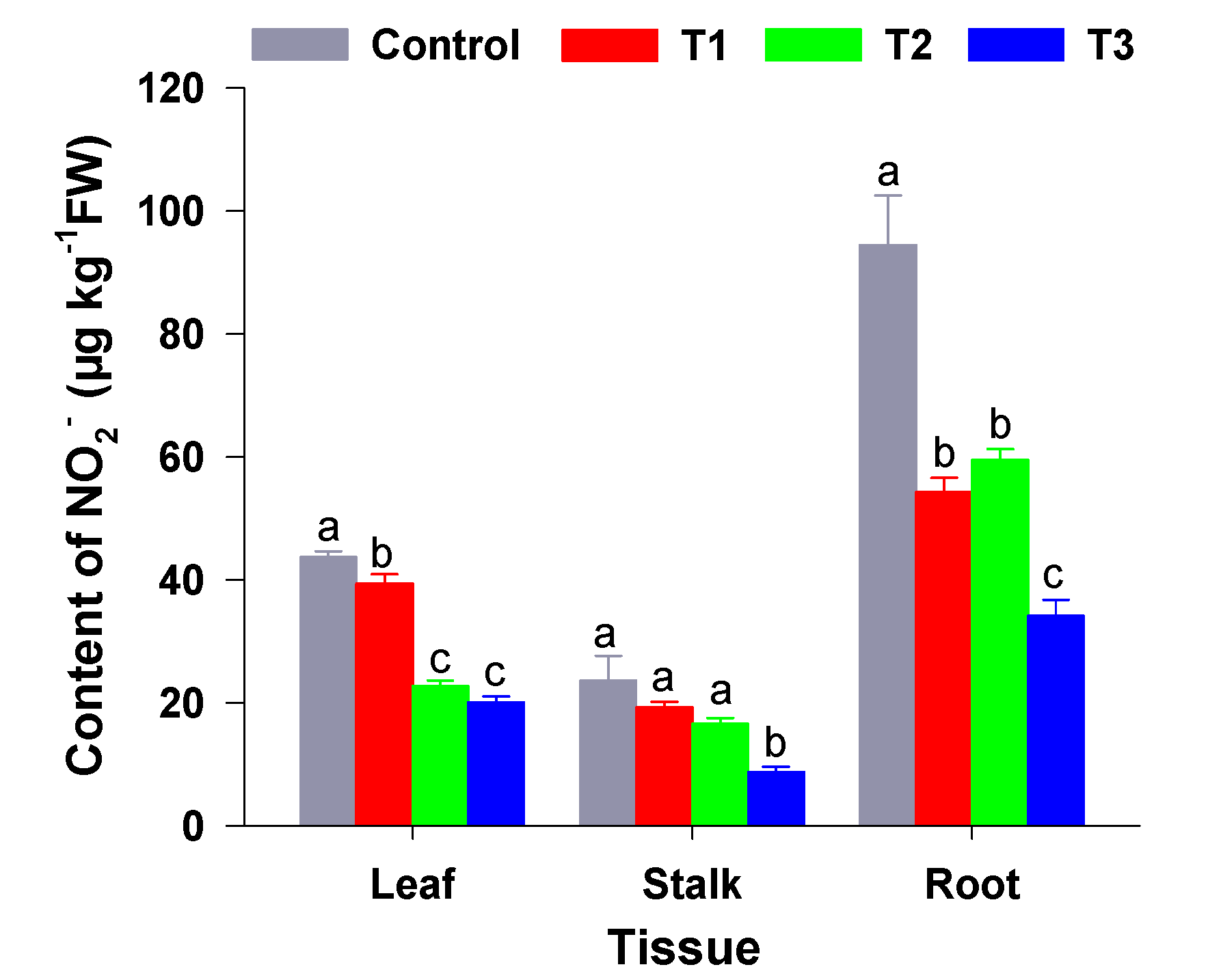

Supplement: Supplementary Figure 1 — NO2– content in root, stem and leaf of flowering Chinese cabbage under different NH4+/NO3– ratios. [file Image_1.tiff]

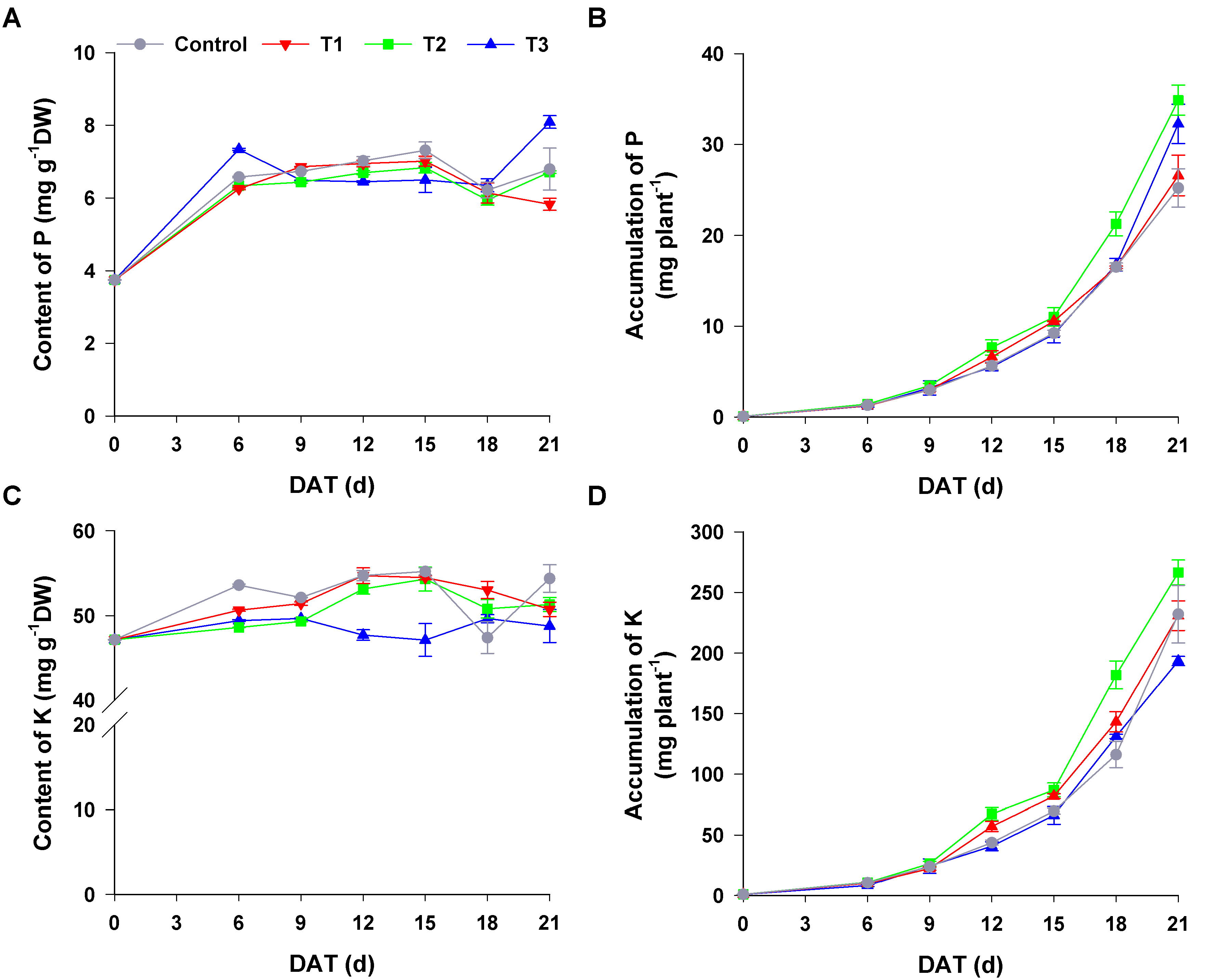

Supplement: Supplementary Figure 2 — Effect of different NH4+/NO3– ratios on the content and accumulation of total P and K in the growth period of flowering Chinese cabbage. [file Image_2.tiff]

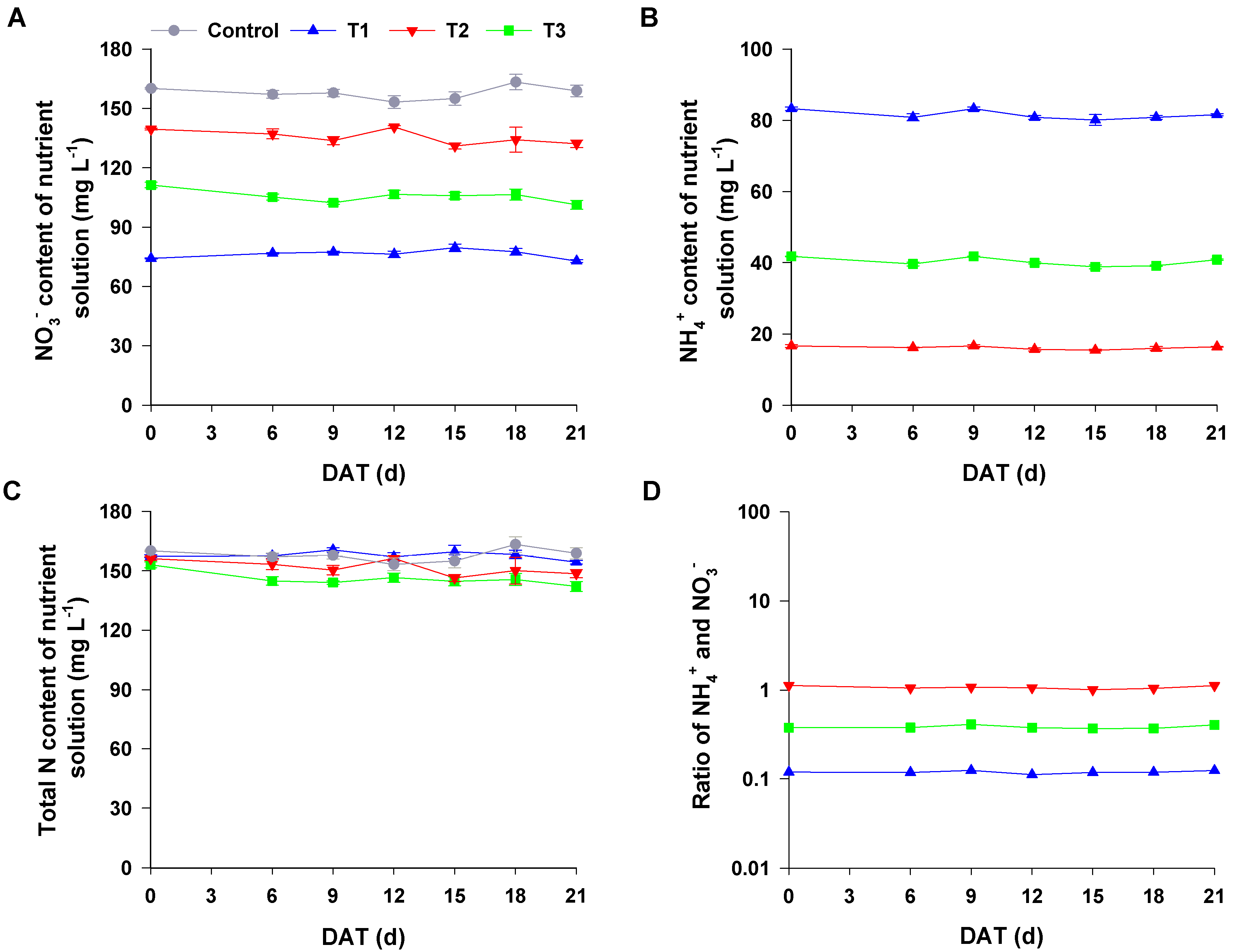

Supplement: Supplementary Figure 3 — The change of NO3–, NH4+ and total N content of the nutrient solution without seedlings under different NH4+/NO3– ratios. [file Image_3.tiff]

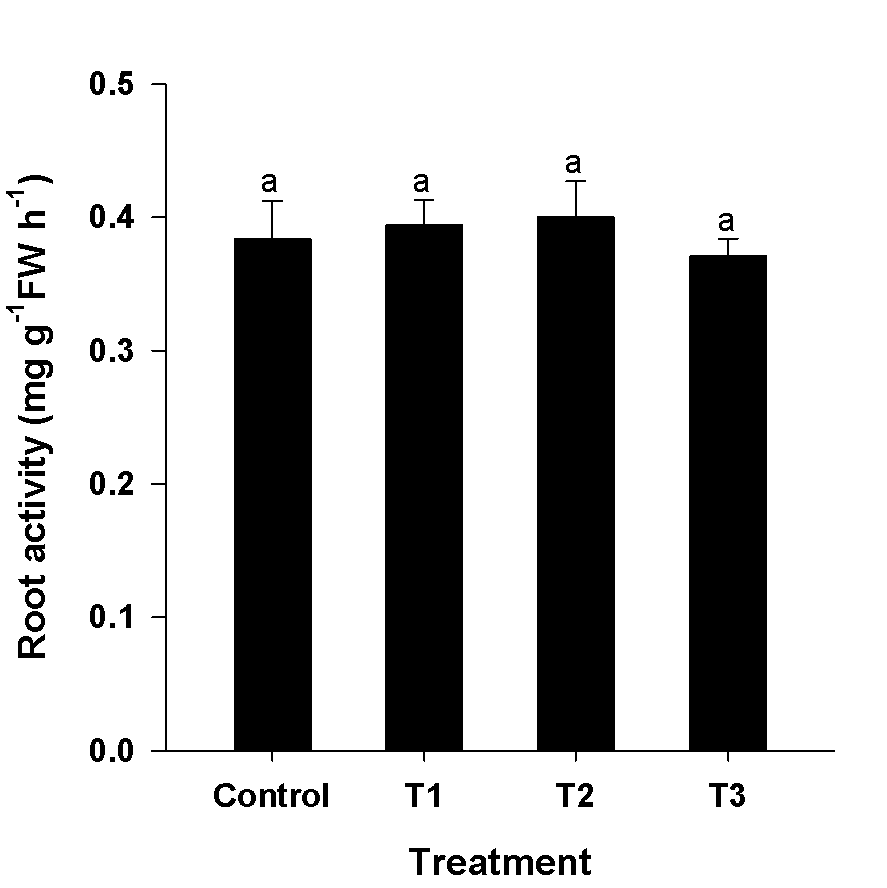

Supplement: Supplementary Figure 4 — The root activity of flowering Chinese cabbage under different NH4+/NO3– ratios. [file Image_4.TIFF]

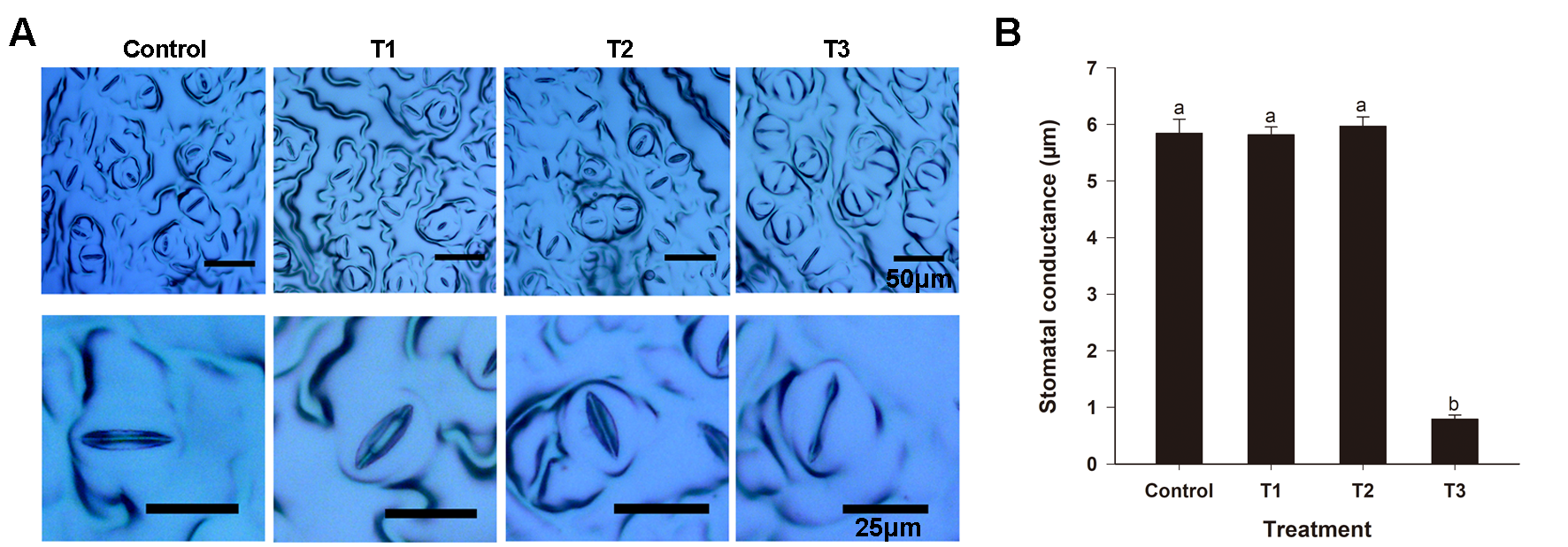

Supplement: Supplementary Figure 5 — Stomatal conductance of leaf in flowering Chinese cabbage in response to different NH4+/NO3– ratios. [file Image_5.tif]
